# G4PromFinder: an algorithm for predicting transcription promoters in GC-rich bacterial genomes based on AT-rich elements and G-quadruplex motifs

**DOI:** 10.1186/s12859-018-2049-x

**Published:** 2018-02-06

**Authors:** Marco Di Salvo, Eva Pinatel, Adelfia Talà, Marco Fondi, Clelia Peano, Pietro Alifano

**Affiliations:** 1 Department of Biological and Environmental Sciences and Technologies, University of Salento, Lecce, Italy; 2 Institute of Biomedical Technologies National Research Council, Milan, Segrate Italy; 3 Department of Biology, University of Florence, Florence, Italy; 4 Institute of Genetic and Biomedical Research (IRGB), UOS of Milan, National Research Council, Milan, Italy; 5 Humanitas Clinical and Research Center, Milan, Rozzano Italy

**Keywords:** G4PromFinder, Promoters, G-Quadruplex, Motif, GC-rich genomes, Promoter elements

## Abstract

**Background:**

Over the last few decades, computational genomics has tremendously contributed to decipher biology from genome sequences and related data. Considerable effort has been devoted to the prediction of transcription promoter and terminator sites that represent the essential “punctuation marks” for DNA transcription. Computational prediction of promoters in prokaryotes is a problem whose solution is far from being determined in computational genomics. The majority of published bacterial promoter prediction tools are based on a consensus-sequences search and they were designed specifically for vegetative σ^70^ promoters and, therefore, not suitable for promoter prediction in bacteria encoding a lot of σ factors, like actinomycetes.

**Results:**

In this study we investigated the possibility to identify putative promoters in prokaryotes based on evolutionarily conserved motifs, and focused our attention on GC-rich bacteria in which promoter prediction with conventional, consensus-based algorithms is often not-exhaustive. Here, we introduce G4PromFinder, a novel algorithm that predicts putative promoters based on AT-rich elements and G-quadruplex DNA motifs. We tested its performances by using available genomic and transcriptomic data of the model microorganisms *Streptomyces coelicolor* A3(2) and *Pseudomonas aeruginosa* PA14. We compared our results with those obtained by three currently available promoter predicting algorithms: the σ^70^consensus-based PePPER, the σ factors consensus-based bTSSfinder, and PromPredict which is based on double-helix DNA stability. Our results demonstrated that G4PromFinder is more suitable than the three reference tools for both the genomes. In fact our algorithm achieved the higher accuracy (F_1_-scores 0.61 and 0.53 in the two genomes) as compared to the next best tool that is PromPredict (F_1_-scores 0.46 and 0.48). Consensus-based algorithms produced lower performances with the analyzed GC-rich genomes.

**Conclusions:**

Our analysis shows that G4PromFinder is a powerful tool for promoter search in GC-rich bacteria, especially for bacteria coding for a lot of σ factors, such as the model microorganism *S. coelicolor* A3(2). Moreover consensus-based tools and, in general, tools that are based on specific features of bacterial σ factors seem to be less performing for promoter prediction in these types of bacterial genomes.

**Electronic supplementary material:**

The online version of this article (10.1186/s12859-018-2049-x) contains supplementary material, which is available to authorized users.

## Background

In all living organisms the flow of genetic information starts with gene transcription, an essential process that is tightly regulated at each step (initiation, elongation, termination). In bacteria and archaea a single RNA polymerase (RNAP) carries out this process, whereas in eukaryotes multiple different RNAP are responsible for transcription of different classes of genes [1]. Despite a lot of differences in transcription machinery among the three domains of life (including RNAP subunit composition with five subunits in bacterial (α2ββ′ω) and more than 12 subunits in archaeal and eukaryotic RNAP), evolutionary conserved features such as similar overall shape in RNAP, highly conserved active centers and similar contact to the nucleic acid chains have been recognized [2, 3]. Structural and functional similarities also extend to several accessory factors modulating the different steps of the transcription cycle.

In bacteria, specific transcription initiation requires sigma (σ) factors that, when bound to RNAP, recognize and melt promoters [4]. Based on sequence, structural and functional similarity, the bacterial σ-factors can be grouped into two families, the σ^70^- and σ^54^-family (this latter existing in most but not all bacteria), with little if any sequence identity between them [5, 6]. In contrast to bacterial RNAP, archaeal RNAP and eukaryotic RNAP II utilizes two key basal factors, transcription factor B (TFB for archaeal RNAP, TFIIB for eukaryotic RNAP II) and TATA-binding protein (TBP) rather than σ factors for transcription initiation. TFB/TFIIB and TBP bind to DNA and subsequently recruit RNAP and additional factors to form a core initiation complex [7, 8]. However, recently, structural comparison of initiating RNAP complexes and structure-based amino acid sequence alignment have provided evidence of structural and functional analogies, and evolutionary relatedness between bacterial σ^70^-family factors and archaeal/eukaryotic TFB/TFIIB suggesting a simple model for promoter evolution and genesis of transcription systems [9, 10]. The model is based on apparent conservation of helix-turn-helix (HTH) motifs in archaeal/eukaryotic TFB/TFIIB and bacterial σ^70^-family factors. These HTH motifs are involved in recognition of the structural promoter elements: a GC-rich “anchor sequence” (corresponding to bacterial − 35 element and archaeal/eukaryotic BRE_up_) and a downstream located “AT-rich element” (corresponding to bacterial − 10 element [TATAAT, Pribnow box] and TATAAAAG boxes). Contact to double strand anchor DNA maintains the position of the most C-terminal HTH domain, while more N-terminal HTH domains facilitate bubble opening and initiation [9, 10].

Recently, G-quadruplex motifs, tertiary structures formed by nucleic acid sequences that are rich in guanine via non-Watson-Crick base pairing, have received a great deal of attention because of their putative role in promoter function [11]. In these dynamic structures, four guanine residues can associate through Hoogsteen hydrogen binding to form a square planar structure called a guanine tetrad, and two or more guanine tetrads can stack on top of each other to form a G-quadrulpex [12–15]. Interestingly, more than 40% of human gene promoters contain one or more G-quadruplex motifs [16]. In fungi G-quadruplex DNA motifs are significantly associated with promoter regions and to a lesser extent with open reading frames (ORFs) [17], and these DNA motifs are more conserved than expected from a random distribution among related fungi suggesting in vivo functions that are under evolutionary constraint [18]. Conserved G-quadruplex DNA motifs have been also reported in promoters of orthologous gene across phylogenetically distant prokaryotes [19], and, very recently, a conserved putative G-quadruplex-Hairpin-Duplex switch has been described [20].

The evolutionary relatedness and/or functional analogies in transcription initiation mechanisms between all three domains of life prompted us to explore the possibility of recognizing promoter elements in prokaryotic genomes based on conserved structured motifs. In particular, we focused our attention on GC-rich bacterial genomes where promoter prediction with conventional, consensus-based algorithms is often difficult and certainly not exhaustive [21]. Promoter prediction is especially problematic in actinomycetes, a group of mycelial organisms with complex transcriptional patterns because their large genomes may encode more than 60 sigma factors [22], although consensus sequences have been proposed for computer assisted promoter identification and classification in *Streptomyces* spp. [23]. In this study we have developed an algorithm to identify putative promoters based on AT-rich elements and G-quadruplex DNA motifs in the GC-rich “anchor sequence”, and tested its performances by using available genomic and transcriptomic data of the model microorganisms *Streptomyces coelicolor* A3(2) [22] and *Pseudomonas aeruginosa* PA14 [24]. Results were compared with those obtained by some currently available tools for bacterial promoter prediction. Currently available tools for prokaryote promoter prediction include BPROM [25], NNPP2 [26], PePPER [27], PromPredict [28] and bTSSfinder [29]. BPROM, NNPP2 and PePPER are tools for prediction of prokaryote promoter elements based on a consensus-sequences search and they were designed specifically for vegetative σ^70^ promoters. bTSSfinder is the most recent consensus-based promoter prediction algorithm in prokaryotes. It extends the concept of consensus prediction to five classes of σ factors in *E. coli* (σ^70^, σ^38^, σ^32^, σ^28^ and σ^24^) and to five classes of σ factors in *Cyanobacteria* (σ^A^, σ^C^, σ^H^, σ^G^ and σ^F^). This tool performed successfully in *E. coli* genome achieving very high accuracy values (*F*_1_-score = 0.93) [29]. PromPredict instead identifies promoter regions on the basis of DNA double helix stability, therefore using a different strategy than consensus-based algorithms. In fact, PromPredict algorithm is based on the general observation that promoter regions are less stable than flanking regions [21, 30]. For this reason, PromPredict is a more general tool than consensus-based tools and could be more suitable in GC-rich bacteria featuring diverse σ factors. For comparison, we focused our attention on PromPredict [31], on the most recent consensus-based tools PePPER [32] and on bTSSfinder [33]. We excluded from the comparison BPROM and NNPP2 because they work similarly to the most recent PePPER. All these tools are designed for a genome-wide prediction. PePPER was optimized for *E. coli*, PromPredict for both *E. coli* and *B. subtilis*, while bTSSfinder for *E. coli* and *Cyanobacteria*.

## Implementation

### Programming language and data sets

G4PromFinder algorithm was implemented in Python (v.3.5) [34], and works as a genome-wide promoter predictor taking as input bacterial genome-sequences. In particular, we used available genomic sequences (from National Center for Biotechnology Information) of the model microorganisms *S. coelicolor* A3(2) (accession code NC_003888.3) and *P. aeruginosa* PA14 (accession code NC_008463.1) (see below) for promoter predictions and their genomic annotation together with transcriptomic data [22, 24] for the prediction quality evaluation. The method to identify putative promoter elements is described below.

### Method to identify putative promoters

**Fig. 1:**
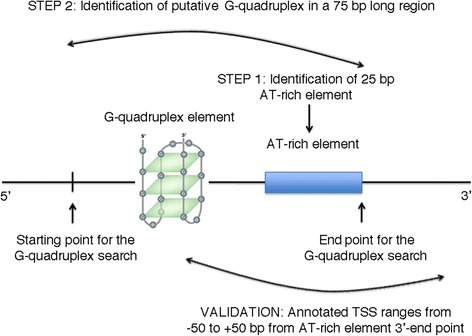
Method used for the prediction and the validation of putative promoters

A two-step procedure was used to detect putative promoters (Fig. 1). The first step consisted in the identification of the putative promoter “AT-rich element”. To this purpose, the algorithm slides a window of 25 bp over the query sequence, 1 bp at a time, until the AT% content of the window reaches the threshold value of 40%. Afterwards, by scanning a window of 75 bp (starting from the position where the threshold value of the AT% content was reached), the 25 bp long region with maximal AT content (herein referred to as AT-rich element) is selected. The second step was the identification of putative G-quadruplex motifs extended up to 50 bp upstream from the 5′-end of the selected AT-rich element. Motif G_x_N_y_G_x_N_y_G_x_N_y_G_x_ with 2 ≤ x ≤ 4, 1 ≤ y ≤ 10 and maximum length of 30 bp is commonly used to predict the presence of G-quadruplexes [35]. G-quadruplexes could have an influence on gene expression also when localized on the reverse strand relative to transcription direction [36]. For this reason we searched for putative G-quadruplex motifs on either sense or antisense strand (motif C_x_N_y_C_x_N_y_C_x_N_y_C_x_ with 2 ≤ x ≤ 4, 1 ≤ y ≤ 10 and maximum length of 30 bp was used to predict putative G-quadruplex on antisense strand). We considered as a single prediction all the predictions that were within 35 bp from each other, because most signals involved in determining TSS are located in the short region between the − 35 and the − 10 boxes. It is also relevant to point out that the length of the regions evaluated in the first step (25 bp) and in the second step (50 bp) are arbitrary; they were determined experimentally to optimize our search, and were compatible with the overall geometry of bacterial RNAP-promoter complex and with the proposed model for the genesis of transcription systems [9, 10]. Finally, two additional features of our algorithm are: i) the possibility of predicting multiple putative promoters in a single query region and ii) the possibility of searching for promoters in both strands.

### TSS global map datasets

To evaluate the reliability of our promoter predictions we used TSS global maps obtained by dRNAseq experiments. For *S. coelicolor* A3(2) 3570 TSSs were identified [22], and were categorized by their positions relative to known coding sequences (CDSs) giving 2771 primary TSSs (P) associated with currently annotated genes, which corresponds to 35.0% of the total genes in the *S. coelicolor* genome. In addition to P, 333 secondary TSSs (S) were identified revealing a total of 297 transcription units initiated by more than one TSS. 256 TSSs mapped in the antisense strand (A) of 241 genes, while 79 internal TSSs (I) were detected within 73 genes. Finally, 131 TSSs were mapped to IRs with no previously associated genes (N). For *P. aeruginosa* PA14, 2117 TSSs were predicted spanning 3325 protein coding genes (55% of all protein coding genes) [24]. In this last study, TSSs were not categorized.

### Generation of the positive and negative sets of sequences

Using the publicly available genomes of *S. coelicolor* A3(2) and *P. aeruginosa* PA14 (accessions above) and the above-indicated TSS annotations, we created, for each of the two genomes, a promoter set (positive set) consisting of 251 bp long sequences covering the region from − 200 bp to + 51 bp with respect to each experimentally annotated TSS. Hence, for *S. coelicolor* A3(2) and *P. aeruginosa* PA14 genomes, the positive set consisted of 3570 and 2117 sequences, respectively.

As a negative set of sequences we considered all the IRs < = 251 bp and > = 50 bp in length in which promoters are not expected. Precisely, we considered all the previous IRs that separated two convergently oriented CDSs. In order to compare regions of same length, we decided to extend IRs < 251 bp equally from both their extremities until the length of 251 bp was reached. Finally, we assessed the total absence of annotated TSSs in the previous regions. For *S. coelicolor* A3(2) genome, the negative set consisted of 548 sequences, while for *P. aeruginosa* PA14 genome it consisted of 338 sequences.

### Evaluation of the performances of the promoter predictor

To estimate the performances of G4PromFinder, we used the following statistical measures:

- Recall (sensitivity or the true positive rate) = TP/(TP + FN)
- Precision (the positive predictive value) = TP/(TP + FP)
- Specificity (the true negative rate) = TN/(TN + FP)
- Accuracy (the fraction of samples correctly classified) = (TP + TN)/(TP + TN + FP + FN)
- F_1_-score (the harmonic mean of Precision and Accuracy) = 2*Precision*Recall/(Precision+Recall)
- where TP = True positives, FP = False positives, FN = False negatives and TN = True negatives.

In accordance with the validation strategies adopted in previous studies [29], we considered a predicted promoter as a true positive (TP) if it started within 50 bp from an experimentally derived TSS (upstream or downstream). It is important to point out that at most one TP was considered for each sequence of the positive set. We considered as false positives (FP) all the samples of the negative set in which the algorithm predicted at least a promoter, as true negatives (TN) the sequences of the negative set in which the algorithm did not predict promoters and, finally, as false negatives (FN) the sequences of the positive set in which TPs were absent.

## Results

### Genome statistics, IRs and promoter prediction with consensus sequence-based algorithm

**Fig. 2:**
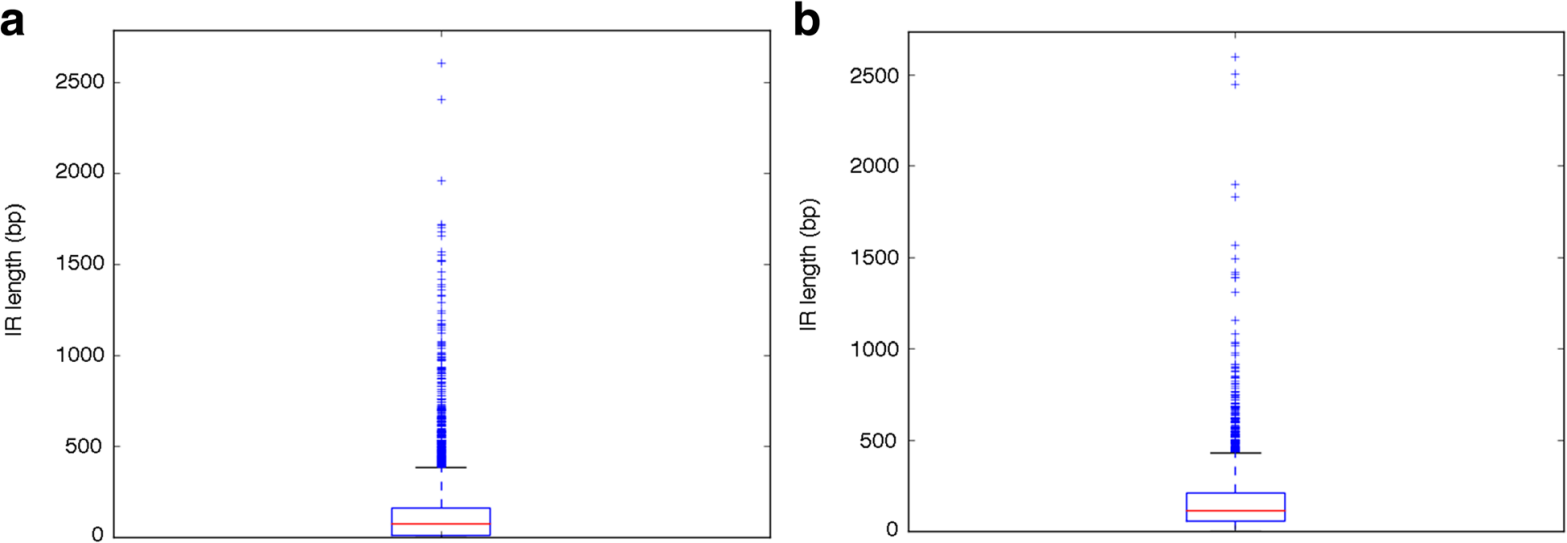
Boxplot of IRs length for *S. coelicolor* (**a**) and *P. aeruginosa* (**b**)

Data sources in this study were: i.) the annotated, whole-genome sequences of *S. coelicolor* A3(2) [37] and *P. aeruginosa* PA14 [38] for promoter prediction; ii) the TSS list obtained by dRNAseq experiments [22, 24] for promoter prediction validation. The complete genome of *S. coelicolor* A3(2) has a GC-content of 72.1% and consists of putative 7825 genes (mean GC-content 72.1%), with the median IR length of 118 bp, the first quartile IR length of 13 bp and the third of 162 bp (Fig. 2). Regarding *P. aeruginosa* PA14, its genome, with a GC-content of 66.3%, consists of 5973 annotated genes (mean GC-content 66.2%) with the median IR length of 118 bp, the first quartile IR length of 61 bp and the third of 211 bp (Fig. 2).

Preliminarily, in all IRs we searched for the presence of the “-35 consensus sequence” (TTGAC for *S. coelicolor* A3(2) and TTGNC for *P. aeruginosa* PA14) and the “-10 consensus sequence” (TANNNT for *S. coelicolor* A3(2) and TANAAT for *P. aeruginosa* PA14) for σ^70^-family factors [23, 39] separated by a sequence ranging in length from 16 to 22 bp. We detected both these sequences only in 1.6% and 0.4% of IRs of *S. coelicolor* A3(2) and *P. aeruginosa* PA14, respectively. This result further shows how difficult is to predict promoters by using consensus-based methods in GC-rich bacterial genomes.

### Promoter prediction by AT-rich element and G-quadruplex motif-based algorithm and evaluation

Statistics of putative promoters that were predicted by G4PromFinder algorithm in the positive set are summarized in Table 1. Preliminarly we considered all the putative promoters predicted by G4PromFinder, without further constraints. G4PromFinder predicted, respectively, for *S. coelicolor* A3(2) and *P. aeruginosa* PA14, putative promoters in almost all the examined regions, precisely in 91.2% and 91.5% of such regions (Table 1). Overall, the algorithm predicted, respectively, 3751 and 2305 putative promoters in the two genomes. Therefore multiple putative promoters were associated with some of the samples. Precisely, in 13.8 and 17.4% of examined regions in *S. coelicolor* A3(2) and *P. aeruginosa* PA14, respectively, more than one predicted promoter could be found.

**Table 1:** Statistics of predicted promoters by G4PromFinder algorithm

Bacterial genome	Positive dataset size	Regions with at least one prediction (%)	Regions with more predictions (%)	Total number of prediction
*Streptomyces coelicolor* A3(2)	3570	91.2	13.8	3751
*Pseudomonas aeruginosa* PA14	2117	91.5	17.4	2305

We evaluated G4PromFinder performances on promoter prediction using a positive sequence set including all regions surrounding by a dRNAseq verified TSS and a negative sequence set composed by short IRs located between two convergently oriented CDSs, for both *S. coelicolor* A3(2) and *P. aeruginosa* PA14 genomes (see Implementation section for details). To fairly compare the positive and negative sets, which originally do not have the same size, we decided to randomly select 548 and 338 regions of the positive sets, respectively in *S. coelicolor* A3(2) and *P. aeruginosa* PA14 genomes, and we repeated the testing 10 times on different series of randomly selected sequences to obtain the mean values reported in Table 2 (column 1 and 2). We observed good performances in both bacterial genomes. In fact the F_1_-scores obtained in *S. coelicolor* A3(2) and *P. aeruginosa* PA14 were 0.61 and 0.53, respectively (Table 2). Recall-values obtained were high, about 70% (precisely 70.1 and 69.0%), while precision-values were lower (54.3 and 43.1%). Interestingly, in *S. coelicolor* A3(2) about 40% of validated promoters contained the “-10 consensus sequence” (TANNNT) that was previously proposed for σ^70^-family factors in Streptomycetes [23] (Table 3). In contrast, only a low percentage of validated promoters (6.1%) contained the “-35 consensus sequence” (TTGAC) for σ^70^-family factors [22] (Table 3). In *P. aeruginosa* PA14, the “-10 consensus sequence” (TANAAT) and the “-35 consensus sequence” (TTGNC) for σ^70^-family factors were contained in 7.4% and 28.2% of validated promoters, respectively (Table 3)*.* Moreover, the mean AT content of the validated promoter AT-rich elements obtained was rather higher than the threshold value of 40% (see Implementation section), 48.5% and 53.3% in *S. coelicolor* A3(2) and *P. aeruginosa* PA14, respectively. These values are also higher than the mean AT content of the total validated promoters (Table 3).

**Table 2:** - Testing results of G4PromFinder^a^

Bacterial genome	TP	FN	FP	TN	Precision (%)	Recall (%)	Specificity (%)	Accuracy (%)	*F*_1_-score
*Streptomyces coelicolor* A3(2)	384	164	324	224	54.3	70.1	40.8	55.5	0.61
*Pseudomonas aeruginosa* PA14	233	105	308	30	43.1	69.0	8.9	38.9	0.53

^a^Test experiments were repeated 10 times for 548 and 338 randomly selected sequences of positive sets of *S. coelicolor* A3(2) and *P. aeruginosa* PA14, and the means were taken

**Table 3:** – Some features of the validated promoters

Bacterial genome	Mean GC content of validated promoters (%)	Mean AT content of the AT-rich element of validated promoters (%)	Validated promoters with “-35 consensus” (%)	Validated promoters with “-10 consensus” (%)
*Streptomyces coelicolor* A3(2)	64.5	48.5	6.1	40.1
*Pseudomonas aeruginosa* PA14	59.6	53.3	28.2	7.4

### A negative control: Specificity control of the promoter element G-quadruplex

In these GC-rich bacterial genomes, the G-quadruplex motif (see Implementation section) occurs very frequently. In fact we found about 120,000 and 70,000 instances of the G-quadruplex motif in *S. coelicolor* A3(2) and *P. aeruginosa* PA14 genomes. For this reason, we decided to carry out a negative control, in order to assess the not-random presence of a G-Quadruplex motif in a promoter. This control consisted in the searching for a random sequence motif with the same frequency as the G-quadruplex motif in the genome, in the identification of putative promoters as AT-rich elements that were preceded by this random sequence motif, and in their subsequent validation by using the same procedure adopted for G4PromFinder prediction. We used as random motif tetranucleotides sequences with a GC content similar to that of the entire genomes, preceded and followed by 13 random bp (in order to have a motif of length similar to G-quadruplex motif). In *S. coelicolor* A3(2) we carried out two controls, each with two pairs of tetranucleotide sequences (GCAG and GCTG; CACG and TCGC) that together have the same frequency to the G-quadruplex motif, while in *P. aeruginosa* PA14 we carried out one control with a pair of tetranucleotide sequences (GACG and ACGC). The random approach achieved lower accuracy (in *S. coelicolor* A3(2), F_1_-score for the first pair of tetranucleotides 0.56, F_1_-score for the second pair of tetranucleotides 0.51; in *P. aeruginosa* PA14 F_1_-score 0.45) compared to G4PromFinder (F_1_-score 0.61 and 0.53 in *S. coelicolor* A3(2) and *P. aeruginosa* PA14, Table 2). From these tests resulted that the fraction of the positive results obtained by G4PromFinder that surely were not a consequence of random chance is 0.12 in *S. coelicolor* A3(2) (1–0.535/0.61; 0.535 mean value of F_1_-scores of the two negative controls) and 0.15 in *P. aeruginosa* PA14 (1–0.45/0.53). The presence of AT-rich element in our negative control is the most probable reason for the relatively high performances in promoter predictions obtained by it. Indeed AT-rich element has by itself a well-known role in promoter regions definition. In any case the random approach achieved lower accuracy compared to G4PromFinder, and we can conclude that the G-quadruplex element has a higher specificity in the association with the AT-rich element compared to a random sequence with the same frequency of G-quadruplex and with a GC-content similar to that of the whole genome.

### Comparison with PePPER, PromPredict and bTSSfinder tools.

We compared our results with those obtained by PePPER [27], PromPredict [28] and bTSSfinder [29] tools. PePPER predicts prokaryote promoters based on a consensus-sequences search. Precisely, PePPER software looks for the “-35 consensus sequence” and “-10 consensus sequence” for σ^70^-family factors of *Escherichia coli* allowing a certain degree of variability for the bases belonging to the consensus sequences. PePPER takes the annotated bacterial genome sequence as input and provides as output the positions of putative TSS, “-35 consensus sequence” and “-10 consensus sequence”, with a score assigned to them that indicates the probability that the extracted region actually corresponds to a promoter. In contrast, PromPredict predicts prokaryote promoters based on differences in DNA double helix stability in promoter and non-promoter regions, taking as input bacterial genome sequences and providing as output promoter coordinates, with a reliability level assigned to them. bTSSfinder, instead, predicts putative promoters for five classes of σ factors in *Cyanobacteria* (σ^A^, σ^C^, σ^H^, σ^G^ and σ^F^) and for five classes of σ factors in *E. coli* (σ^70^, σ^38^, σ^32^, σ^28^ and σ^24^) taking as input bacterial genome sequences and providing as output TSS coordinates [29]. Preliminarily we run the three comparison tools on the whole genome of *S. coelicolor* A3(2) and *P. aeruginosa* PA14 considering all the identified promoter regions independently from their score. Table S1 (in Additional file 1) shows the global numbers of prediction obtained by each tool in comparison to G4PromFinder whole genome predictions. Then we intersected the three tool genome wide predictions with the positive and negative region sets already used for the evaluation of G4PromFinder performances (see Implementation). We considered as positive intersections only the predicted promoters falling for their entire length within those regions (i.e. the predicted promoters by PePPER and PromPredict whose coordinates falling within those regions and all the predicted TSSs by bTSSfinder falling within those regions). We defined true promoters those whose position difference between the experimentally derived TSS and the predicted promoter regions was included in the range ± 50 bp, as we have done for G4PromFinder.

The results for *S. coelicolor* A3(2) and *P. aeruginosa* PA14 are shown in Table 4. We evaluated the performances of the four methods by using precision, recall and F_1_-score. This comparison clearly indicates that G4PromFinder has significantly higher prediction accuracy, both in *S. coelicolor* A3(2) and in *P. aeruginosa* PA14. In fact, as presented in Table 4, G4PromFinder produced the best performance (F_1_-score 0.61 in *S. coelicolor* A3(2), F_1_-score 0.53 in *P. aeruginosa* PA14), followed by PromPredict (F_1_-score 0.46 in *S. coelicolor* A3(2), F_1_-score 0.48 in *P.aeruginosa* PA14), bTSSfinder for *E. coli* σ factors (F_1_-score 0.38 in *S. coelicolor* A3(2), F_1_-score 0.36 in *P.aeruginosa* PA14), and finally bTSSfinder for *Cyanobacteria* σ factors (F_1_-score 0.28 in *S. coelicolor* A3(2), F_1_-score 0.28 in *P.aeruginosa* PA14) and PePPER (F_1_-score 0.32 in *S. coelicolor* A3(2), F_1_-score 0.42 in *P.aeruginosa* PA14). Therefore consensus-based algorithms (PePPER and bTSSfinder) produced lower performances with the analyzed GC-rich genomes. Actually PePPER algorithm provided the highest precision values, but it also produced the lowest recall values and, for this reason its F_1_-scores were very low.

**Table 4:** Comparison between G4PromFinder, PePPER, PromPredict and bTSSfinder testing results^a^

Tools	Bacterial genome	*Streptomyces coelicolor* A3(2)	*Pseudomonas aeruginosa* PA14
G4PromFinder	Recall	0.70	0.69
	Precision	0.54	0.43
	F_1_-score	0.61	0.53
PePPER	Recall	0.20	0.31
	Precision	0.78	0.67
	F_1_-score	0.32	0.42
PromPredict	Recall	0.51	0.56
	Precision	0.41	0.42
	F_1_-score	0.46	0.48
bTSSfinder (for *E. coli* σ factors)	Recall	0.45	0.41
	Precision	0.33	0.31
	F_1_-score	0.38	0.36
bTSSfinder (for *Cyanobacteria* σ factors)	Recall	0.29	0.30
	Precision	0.27	0.26
	F_1_-score	0.28	0.28

^a^Test experiments were repeated 10 times for 548 and 338 randomly selected sequences of positive sets of *S. coelicolor* A3(2) and *P. aeruginosa* PA14, and the means were taken

Moreover, we carried out another comparison between G4PromFinder and the available promoter prediction programs. To perform this analysis, we considered all the predicted promoters by the four programs that were within the regions of the positive set, considering now as false positives the predictions whose distance from the annotated TSS was more than 50 bp. The results of the comparison are presented in Table 5, and again they clearly show that G4PromFinder has the highest prediction accuracy in these bacterial genomes.

**Table 5:** Comparison between G4PromFinder and available promoter prediction programs assessed on all the samples of the positive sets

	Program	TP	FN	FP	precision	recall	F_1_-score
*S. coelicolor* A3(2)	G4PromFinder	2850	870	901	0.76	0.76	0.76
	PromPredict	2075	1582	934	0.69	0.56	0.62
	PePPER	1538	2768	683	0.69	0.35	0.47
	bTSSfinder (*E. coli*)	1449	2121	974	0.59	0.40	0.48
	bTSSfinder (Cyanob.)	1166	2404	1151	0.50	0.32	0.39
*P. aeruginosa* PA 14	G4PromFinder	1682	563	623	0.73	0.74	0.74
	PromPredict	1351	813	549	0.71	0.62	0.66
	PePPER	2015	1383	954	0.67	0.59	0.63
	bTSSfinder (*E. coli*)	923	1194	497	0.65	0.43	0.52
	bTSSfinder (Cyanob.)	687	1430	685	0.50	0.32	0.39

**Fig. 3:**
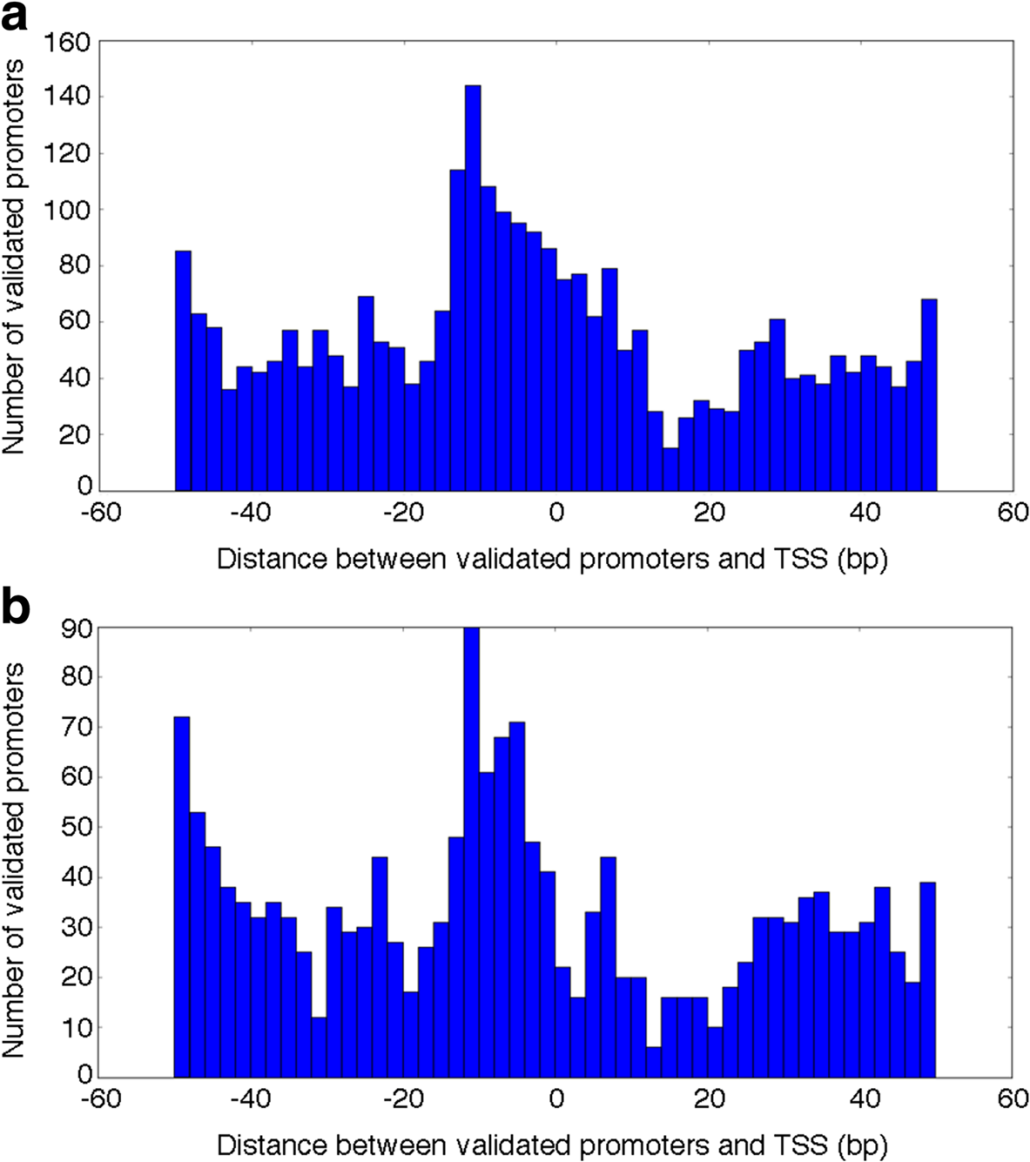
Distribution of validated promoters in *S. coelicolor* A3(2) (**a**) and *P. aeruginosa* PA14 (**b**) as a function of their distance from the TSSs obtained by dRNAseq experiments and used for validation.Predicted promoters are grouped based on distances between the AT-rich element 3′-end points and the annotated TSS. A: predicted promoters in *S. coelicolor* A3(2); B: predicted promoters in *P. aeruginosa* PA14

In Fig. 3 we, instead, show the distributions of distances occurring between the 3′-end points of validated promoters of G4PromFinder and the TSSs used for validation. In all examined cases, we noticed a peak of distribution around the “-10” value.

## Discussion and conclusions

In this study we investigated the possibility of predicting prokaryotic promoters by detecting evolutionarily conserved motifs. We focused on possible G-quadruplex structures upstream of AT-rich elements. The rationale started from the evidence that in human, yeast and bacterial genomes G-quadruplexes are overrepresented in promoter-proximal regions [18, 19, 40, 41]. In this study we showed that an AT-rich element preceded by a G-quadruplex motif is within ±50 bp from an experimentally identified TSS in 75.6 and 73.4% of total cases, in *S. coelicolor* A3(2) and *P. aeruginosa* PA14 genomes, respectively (Table 5). These high percentages support the idea that G-quadruplex is a prototypical motif involved in general promoter function/regulation.

G-quadruplex are highly dynamic structures whose thermal stability is affected by a number of features including the number of G-quartets present in the structure, the length and the composition of the loops formed by non-guanine bases [42]. Many G-quadruplex DNA structures, once folded, are more thermodynamically stable than double-strand DNA in vitro, and their unfolding kinetics are much slower than those of DNA or RNA hairpin structures [43]. As G-quadruplexes are likely to inhibit DNA and RNA metabolism, their formation must be regulated, and recently, a number of proteins that specifically regulate G-quadruplex folding and unfolding have been identified [41].

There is evidence that G-quadruplex formation in promoter “anchor” (− 35 sequence) elements could impair transcription initiation by RNA polymerase, or if present in the antisense strand of bacterial σ^70^ promoter between “anchor” and “AT-rich” (− 10 sequence) element could impair the initiation-elongation transition (the so-called promoter clearance) [11, 36]. On one hand, recognition of double strand “anchor” sequence in promoters may be strongly influenced by G-quadruplex that could create a physical barrier that hinders RNAP binding or complicates promoter recognition by σ factors. On the other hand, RNAP binding might also facilitate G-quadruplex formation on antisense strand after promoter melting, which ultimately might hamper the initiation-elongation transition [36]. Regulation of G-quadruplex folding and unfolding by G-quadruplex-binding proteins might represent a general mechanism to modulate promoter activity.

Noticeably, less than half of validated promoters that were identified by the algorithm in *S. coelicolor* A3(2) genome contained the “-10 consensus sequence” (TANNNT) (Table 3) that was previously proposed for σ^70^-family factors in Streptomycetes [23]. In contrast, a very small percentage of putative promoters in *S. coelicolor* contained the proposed “-35 consensus sequence” (TTGAC) in these bacteria [23] (Table 3). This finding, which may be explained by the occurrence of a huge number of σ-factors in Streptomycetes, confirms how difficult is to identify promoters in Streptomycetes with conventional, consensus-based algorithms.

The evaluation of G4PromFinder performances on *S. coelicolor* A3(2) and *P. aeruginosa* PA14 show high recall values 70.1% and 69.0% respectively (Table 2), but also low specificity values. This is particularly striking for *P. aeruginosa* PA14 genome whose specificity results only 8.9%, (Table 2) if compared to that obtained in *S. coelicolor* A3(2) genome 40.8% (Table 2). We would have expected a lower specificity in *S. coelicolor* A3(2), where the G-quadruplex motif has a higher density (see Results section). This result instead could suggest that G4PromFinder specificity could be linked to the genome GC richness, because the GC-content of *S. coelicolor* A3(2) (72.1%) is higher than that of *P. aeruginosa* PA14 (66.3%). Also at the genome-wide level (Additional file 1: Table S1) we can see number of predictions higher than the number of annotated TSSs but, compared to the other tools, G4PromFinder shows numbers of predictions of the same order of magnitude. The only exception is PePPER which seems the most restricitive. The high number of predictions is probably due to multiple causes, such as, for example, the lack of complete and precise TSS maps and the existence of unknown repression mechanisms for which some computationally predicted promoters are not used in vivo.

The comparison of G4PromFinder predictions with those obtained by PePPER [27], PromPredict [28] and bTSSfinder [29] tools, highlighted also its reliability. Indeed our analysis showed that G4PromFinder produces the best performances in both the genomes, obtaining as F_1_-score 0.61 and 0.53 in *S. coelicolor* A3(2) and *P. aeruginosa* PA14, compared to the next best tool PromPredict (F_1_-score 0.46 and 0.48 in *S. coelicolor* A3(2) and *P. aeruginosa* PA14). The σ factors consensus-based tool bTSSfinder and especially the consensus-based PePPER, which was designed specifically for vegetative σ^70^ promoters, achieved the lowest accuracy (Table 4). This is a further confirmation that a promoter prediction with conventional, consensus-based algorithms is often difficult in this type of bacteria, especially in bacteria coding for several σ factors, like actinomycetes. In fact PePPER, despite achieved the highest precision values, identified very few promoters in the examined genomes; as consequence its recall values were very low (0.20 and 0.31, Table 4). Even bTSSfinder, that in *E. coli* achieved the highest accuracy (F_1_-score 0.93 [29]) compared to the available tools, failed in these genomes. The same results were obtained also when we tested G4PromFinder and the three comparison tools only on the sequences of the positive set (Table 5). G4PromFinder produced the best results and PromPredict was the best of the three available tools used for comparison. Compared to the results reported in Table 4, the only difference was a general increase in F_1_-scores.

On the basis of these findings, we believed that G4PromFinder is a very powerful tool in GC rich bacterial genomes when compared to currently available tools, which are instead suitable in predicting promoter regions in other genomes, especially *E. coli* for which they were optimized.

## Additional files

Additional file 1: Statistics of predicted promoters by G4PromFinder, PromPredict, PePPER and bTSSfinder in the whole genomes of *S. coelicolor* A3(2) and *P. aeruginosa* PA14. (DOCX 13 kb)

Additional file 2: Coordinates of the validated promoters identified by G4PromFinder in *S. coelicolor* A3(2). (XLSX 100 kb)

Additional file 3: Coordinates of the validated promoters identified by G4PromFinder in *P. aeruginosa* PA14. (XLSX 63 kb)

Additional file 4: G4PromFinder algorithm. (PY 8 kb)

## Abbreviations

A: Antisense TSS
CDS: Coding sequence
dRNAseq: Differential RNAseq
FN: False negatives
FP: False positives
HTH: Helix-turn-helix
I: Internal TSS
IR: Intergenic region
N: TSS with no previously associated genes
ORF: Open reading frame
P: Primary TSS
RNAP: RNA polymerase
S: Secondary TSS
TBP: TATA-binding protein
TFB: Transcription factor B for archaeal RNAP
TFIIB: Transcription factor B for eukaryotic RNAP II)
TP: True positives
TSS: Transcription start site
σ factor: Sigma factor
