# Supplementary material for: G4PromFinder: an algorithm for predicting transcription promoters in GC-rich bacterial genomes based on AT-rich elements and G-quadruplex motifs
Source: BMC Bioinformatics. 2018 Feb 6;19:36. doi: 10.1186/s12859-018-2049-x (PMC5801747; doi:10.1186/s12859-018-2049-x)
Supplement: Supplementary file 1 — Statistics of predicted promoters by G4PromFinder, PromPredict, PePPER and bTSSfinder in the whole genomes of S. coelicolor A3(2) and P. aeruginosa PA14. (DOCX 13 kb) [file 12859_2018_2049_MOESM1_ESM.docx]

|  | **Program** | **Total number of predictions** | **% of annotated TSSs that were predicted** | **% of predictions in IRs** | **% of predictions in CDSs** |
| --- | --- | --- | --- | --- | --- |
| ***S. coelicolor* A3(2)** | **G4PromFinder** | 58601 | 75.6 | 23.0 | 77.0 |
|  | **PromPredict** | 54732 | 55.7 | 19.2 | 80.8 |
|  | **PePPER** | 9408 | 22.5 | 60.8 | 39.2 |
|  | **bTSSfinder (*E. coli*)** | 35207 | 40.6 | 16.7 | 83.3 |
|  | **bTSSfinder (Cyanob.)** | 41561 | 32.7 | 12.9 | 87.1 |
|  |  |  |  |  |  |
| ***P. aeruginosa* PA14** | **G4PromFinder** | 53209 | 73.4 | 14.1 | 85.9 |
|  | **PromPredict** | 40194 | 61.6 | 16.7 | 83.3 |
|  | **PePPER** | 16031 | 34.7 | 62.0 | 38.0 |
|  | **bTSSfinder (*E. coli*)** | 27709 | 43.6 | 14.6 | 85.4 |
|  | **bTSSfinder (Cyanob.)** | 31807 | 32.4 | 10.4 | 89.6 |

**Table S1.** Statistics of predicted promoters by G4PromFinder, PromPredict, PePPER and bTSSfinder in the whole genomes of *S. coelicolor* A3(2) and *P. aeruginosa* PA14.
